# Brand equity and willingness to pay for condoms in zimbabwe

**DOI:** 10.1186/1742-4755-8-29

**Published:** 2011-10-26

**Authors:** W Douglas Evans, Noah Taruberekera, Kim Longfield, Jeremy Snider

**Affiliations:** 1 The George Washington University, School of Public Health and Health Services, 2175 K Street, NW, Suite 700, Washington, DC 20037, USA; 2 PSI-Zimbabwe, 30 The Chase West, Emerald Park Offices, Block E, Emerald Hill, Harare, Zimbabwe; 3 Population Services International, 1120 19th Street, NW, Suite 600, Washington, DC 20036, USA

## Abstract

**Background:**

Zimbabwe suffers from one of the greatest burdens of HIV/AIDS in the world that has been compounded by social and economic instability in the past decade. However, from 2001 to 2009 HIV prevalence among 15-49 year olds declined from 26% to approximately 14%. Behavior change and condom use may in part explain this decline.

PSI-Zimbabwe socially markets the Protector Plus (P+) branded line of condoms. When Zimbabwe converted to a dollar-based economy in 2009, the price of condoms was greatly increased and new marketing efforts were undertaken. This paper evaluates the role of condom marketing, a multi-dimensional scale of brand peceptions (brand equity), and price in condom use behavior.

**Methods:**

We randomly sampled sexually active men age 15-49 from 3 groups - current P+ users, former users, and free condom users. We compared their brand equity and willingness to pay based on survey results. We estimated multivariable logistic regression models to compare the 3 groups.

**Results:**

We found that the brand equity scale was positive correlated with willingness to pay and with condom use. Former users also indicated a high willingness to pay for condoms. We found differences in brand equity between the 3 groups, with current P+ users having the highest P+ brand equity. As observed in previous studies, higher brand equity was associated with more of the targeted health behavior, in this case and more consistent condom use.

**Conclusions:**

Zimbabwe men have highly positive brand perceptions of P+. There is an opportunity to grow the total condom market in Zimbabwe by increasing brand equity across user groups. Some former users may resume using condoms through more effective marketing. Some free users may be willing to pay for condoms. Achieving these objectives will expand the total condom market and reduce HIV risk behaviors.

## Introduction

### Background

Zimbabwe suffers from one of the greatest burdens of HIV/AIDS in the world. Recent estimates from the Joint United Nations Program on HIV/AIDS (UNAIDS) indicate that approximately 1.3 million adults 15 years and older were living with HIV/AIDS in 2007 [1]. Zimbabwe has a generalized HIV/AIDS epidemic with HIV transmitted primarily through heterosexual contact and mother-to-child transmission. Key populations at higher risk including migrant laborers, sex workers, girls involved in intergenerational sexual relationships, serodiscordant couples, and members of the uniformed services warrant special attention in the fight against HIV/AIDS. Additionally, lifestyle factors such as multiple concurrent partnerships are a major focus of HIV prevention programming in Zimbabwe [2]. Young adults and women are hardest hit by the epidemic. In 2007, approximately 595,000 women over the age of 15 were estimated to be living with HIV/AIDS in Zimbabwe [3].

Compounding the effects of HIV/AIDS, from 2001 to 2009, Zimbabwe's economy was characterized by hyperinflation and rapid currency devaluation, with associated deterioration in infrastructure, services, employment and purchasing power [4]. Concurrent with this economic free-fall, Zimbabwe's once robust health system has deteriorated with chronic shortages of public health personnel and essential drugs. In 2008, the majority of provincial and district hospitals and clinics virtually stopped operations as a result of the crumbling social infrastructure. In an effort to halt this slide, in February 2009 the government liberalized trade and introduced the US dollar as the official hard currency; the South African Rand and Botswana Pula are also prevalent forms of legal tender [4]. While there is no official data available, dollarization appears to have stabilized the economy, with inflation greatly reduced if not eliminated, most stores are consistently stocked with consumer goods, and prices of many products declining since February, 2009 (some core consumer staples such as cooking oil and flour have declined in price by 60%+) [4].

In this context, social marketing of condoms as an important strategy to combat the spread of HIV/AIDS faces significant hurdles. Despite the severity of the epidemic, prevalence rates in Zimbabwe have shown signs of decline, from 26.0 percent prevalence among adults ages 15 to 49 in 2001 to 15.3 percent prevalence in the same age group in 2007 (UNAIDS, 2008), and then to 14.2% in 2009 [3,5]. Dr. Peter Piot, head of UNAIDS at the time, said that in Zimbabwe, "The declines in HIV rates have been due to changes in behavior, including increased use of condoms, people delaying the first time they have sexual intercourse, and people having fewer sexual partners" [5].

PSI http://www.psi.org is a non-governmental organization (NGO) that socially markets disease prevention and health promotion products and services. To promote safe sexual behavior, PSI-Zimbabwe markets the *Protector Plus (P+) *branded line of condoms. At the time of dollarization in late 2009, P+ was sold at the equivalent of less than $.01USD, representing a virtually free commodity. While such a low price makes the P+ condom highly affordable, it may lead to inefficient allocation of social marketing and public sector resources by excluding commercial products from the marketplace, offering little or no profit incentive for retailers, risking wastage in the distribution system, and smuggling of the product to other countries with higher prices [6]. Moreover, an overly low price may reduce consumers' perceptions of value in the condom product, harming the brand and efforts to market it and increased utilization of condoms overall [7].

PSI-Zimbabwe's objectives in marketing *P+ *are the following: 1) Distribution of an affordable brand of condoms in traditional and non-traditional outlets, and those located in areas where high risk behavior takes place (condom social marketing); 2) Promotion of condom use among groups at high risk and sexually active youth; 3) Developing behavior change communication programs that address the dynamics of high-risk behavior among target groups.

The role of the *P+ *brand is to grow the total market by increasing condom use among sexually active men and women. The brand therefore has a very important role in helping destigmatize condom use and to making it something "everybody is doing" and "everyone is talking about" because of its different presentations and characteristics. Brand equity represents the value that the brand holds for consumers. PSI-Zimbabwe's goal is to measure current brand equity in its *P+ *lines, actively manage brand equity, and increase equity through future brand repositioning, marketing, and management.

PSI-Zimbabwe increased the price of *P+ *condoms to 10 cents in November 2009 (prices noted throughout are US currency). Immediately following this price increase, as with previous price increases, there was an observed steep decline in sales, from over 5 million units sold in October 2009 to just over 2 million units sold in November 2009. There was a rebound in sales to over 3 million in December 2009, but the question facing PSI-Zimbabwe faced was about the current brand equity of *P+ *and how the brand should be actively managed at the new price and in a changing overall marketplace.

### Protector Plus Brand Marketing

An in-depth audience profile was created to provide an illustrative description of the target audience to inform the intervention's communications strategy and activity planning. An 'archetype' of the targeted condom consumer was developed as a guide to the brand marketing. 'Mike' is a 25 year old single man living in with his extended family in Chitungwiza (a small town). He is an informal trader with secondary education and makes an average of $80 per month. He owns and listens to the radio and rides a kombi (shared taxi) to and from his trading place in the city centre. Mike dreams of having a steady girlfriend and providing for his extended family's material needs. He drinks opaque beer and occasionally clear beer whilst hanging out with his numerous friends at the shopping centre during weekends. He worries about HIV infection so he openly discusses condoms with his friends and they encourage each other to use condoms although Mike does not use condoms consistently with his partner.

The P+ brand is positioned as follows to reach its audience: "For Mike, Protector Plus is the condom that gives him control of a demanding lifestyle by reducing the number of things he has to be concerned about." Marketing efforts seek to communicate two key brand attributes to the audience: 1) Protector Plus is easy to use, strong, reliable, and effective and gives me control to enjoy life - Always use Protector Plus (brand promise); 2) I am confident I can use Protector Plus correctly and consistently with my partner - Use Protector Plus every time (self-efficacy).

P+ marketing efforts seek to: 1) Increase the percentage of men ages 20 -30 who believe Protector Plus condoms are reliable, strong and effective; and 2) Increase the percentage of men ages 20 -30 who are capable of using condoms correctly and consistently with all partners.

Additionally, marketing efforts aim to increase positive brand perceptions and self-efficacy to use condoms among young men in the P+ target audience [7]. The theory underlying this marketing strategy is that men are more likely to use P+ if they believe that the condoms are easy to use, reliable, strong and effective [8]. Moreover, inconsistent condom users are more likely to become consistent users of Protector Plus male condoms if they know that they are able to use condoms correctly and are able to talk to every partner about the importance of using condoms.

### Branding and Brand Equity

Brands have been defined as "a set of associations linked to a name, mark, or symbol associated with a product or service" [9]. Thus the significance of brands is in the *associations *they represent, and the resulting behavior (buying a product, engaging in a behavior, maintaining a relationship with the brand) they can engender. In this respect, brands are very much like reputations - they precede the individual or organization and shape how the world responds.

Brand equity is what the brand stands for in the hearts and minds of consumers, and as a metric it captures their identification with and intentions to purchase, use, and engage with the brand [10,11]. It is a primary driver of product and behavioral choice, especially for health commodities with little functional attribute differentiation (e.g., what tangible functions they perform), such as condoms. Given that most purchase decisions are made within the market context of various brand choices (e.g., a luxury brand of condoms, inexpensive, or perhaps free), marketers need reliable measures of brand equity in order to understand consumers' purchase decisions [12].

Brand equity has been previously studied as a mediator, or mechanism by which marketing efforts promote product use and behavior change [13]. Social marketing interventions develop, promote and create awareness about a brand that may refer to a behavior, campaign or product. Brand equity consists of multiple dimensions, including perceptions of quality, loyalty and perceived value, which, when measured, can guide decision-making early in an intervention particularly in evaluating initial promotional efforts. Over time, brand equity would be expected to influence behavior and as such is a potentially useful component in segmentation analyses and an essential component of outcome evaluations [14]. PSI has used this approach with many of its socially marketed brands [15]. In this study, we examine both the role of P+ brand equity and its potential to help in growing the total market for condoms and evaluate the effectiveness of branding in promoting overall condom use as an HIV prevention behavior.

### Current Research

We asked the following specific research questions: 1) What is the current brand equity in *P+ *and other in-market brands? 2) What differences are there in P+ and other in-market brand equity between specific audience segments and brand attributes? 3) Is higher brand equity in P+ and other in-market brands associated with willingness to pay higher prices for condoms overall or for specific brands? The long-term study objective is to establish a baseline of brand equity for future P+ and condom use monitoring and evaluation studies. In this research, we seek to identify opportunities to improve *P+ *brand equity by addressing specific audience segments and brand attributes. We evaluate the effect of the price increase and examine the relationship between product price points and brand equity. In this way, social marketers can consider whether former *P+ *users who have switched or lapsed from condom use would be willing to use condoms or P+ again. Study results also inform decisions about how to grow the total condom market in Zimbabwe by establishing appropriate prices for socially marketed products that would permit commercial brands to enter the market in the future.

## Methods

### Study Design

We conducted interviews with 890 Zimbabwean men in November and December 2010. Study participants were recruited by simple random selection and were a nationally representative sample of Zimbabwean men aged 18-49 residing in rural and urban areas. The sample was stratified to include men who reported having used P+ condoms within the past 3 months (defined as current P+ users), men who had used P+ condoms within the past year but not in the past 3 months (defined as lapsed users), and men who had used public sector condoms in the past 3 months but not the P+ brand.

Additionally, the study included a qualitative study arm in which 30 Zimbabwean men meeting the same recruitment criteria and not included in the quantitative study arm were interviewed in an open-ended, in-depth interview (IDI) format. The IDI form included the same topics as the questionnaire. Results of the qualitative arm will be reported in a future publication.

### Data Collection

For the quantitative arm of the study a two stage cluster sampling strategy was utilized to select eligible participants. The first stage involved selection of Enumeration areas (EAs) from a list of EAs from the 2002 Zimbabwe national population census. Selection of EAs was dependent upon the size of the EA. A list of households for each of the selected EAs was compiled and became the basis for selecting households. A random selection procedure was employed to sample households for study recruitment. Trained interviewers screened participants following a study recruitment criteria. In the selected households, a Kish grid was used to select one participant in cases where the number of eligible respondents was more than one. After providing informed consent individually, participants completed a face-to-face interview following a structured, 45-item questionnaire. The questionnaire was programmed on a personal digital assistant (PDA) device and the interviewer entered responses directly into the PDA data form. The questionnaire took approximately 20 minutes to administer and a total of 890 individual interviews were completed for this study.

The qualitative arm of the study comprised in-depth interviews (IDIs) with randomly selected 7 to 8 participants among *P+ *current users, public sector users, lapsed *P+ *users, and former users. The IDIs explored the same topics examined in the survey, but in the form of open-ended questions with probes and opportunities to explore the participants' reasons for their indicated level of brand equity and other measures. The sample was chosen based on previous experience in similar studies. A total of 30 in-depth interviews were conducted.

### Measures and instruments

We developed and fielded an interviewer-administered, 45-item questionnaire that included items on participant socio-demographics, media use, exposure to condom brand marketing, brand equity, willingness to pay for condoms, and use of specific condom brands. The following are the brand equity scales. Each item was asked on a 4-point scale from 'strongly agree' to 'strongly disagree' with a 'don't know' option. Each scale was asked for each of the brands evaluated in this study (P+ and public sector) for all respondents across the 3 sample strata.

#### Brand loyalty

1) I will use this brand the next time I use a condom; 2) I would wear a baseball cap with this brand name and logo on it; 3) I would recommend my friends use this brand; 4) This is the best brand of condoms; 5) This brand has the best condom advertisements; 6) I would recommend my friends use this brand.

#### Brand quality

1) This brand will not break during sex; 2) This brand gives a pleasant sensation during sex; 3) This brand does not smell like latex; 4) This brand has nice colors; 5) This brand feels good to the touch; 6) This brand is not too thick; 7) This brand is there when I need it; 8) This brand is available at an outlet near me; 9) This brand has sufficient lubricant.

#### Brand leadership/popularity

1) P+ is becoming more popular with people like me; 2) P+ is for people like me; 3) P+ is the leading brand of condoms.

#### Brand value

1) There are good reasons to buy this brand instead of other condom brands; 2) This brand is a good value for the price; 3) This brand is a better value than other condoms.

#### Brand personality

1) People who use this brand are strong; 2) People who use this brand are confident; 3) People who use this brand have a lot of freedom; 4) People who use this brand have fun; 5) People who use this brand find it easy to have girlfriends; 6) People who use this brand are just like me; 7) People who use this brand are like the people that I hang out with.

#### Market Barriers to Brand Use

1) This brand costs more than I would expect; 2) I would be willing to pay more than the current price for this brand; 3) If the price were reduced by 3 cents, I would buy this brand; 4) If the price were reduced by 6 cents, I would buy this brand.

Additionally, we asked participants how much they would be willing to pay for P+ brand condoms. We asked whether they would be willing to buy P+ at 6 levels of price including the actual price (10 cents) and ranging from free to 15 cents per pack of 3 condoms. We examined the relationship between brand equity and willingness to pay.

We also asked about awareness of brands available for sale and free in Zimbabwe. The multivariable regression analyses reported below included 4 co-variates: age, education, marriage status, and socio-economic status. Age was self-reported in years. Education was self-reported by category with 'completed university' as highest. Marriage status included all potential (co-)habitation status. Socio-economic status (SES) was measured by a list of household amenities which in combination would be indicative of wealth relative to the local economy.

### Data Analysis

Stata version 11 (College Station, TX) was used for all statistical analyses. Survey Participants were grouped into three categories - Current P+ User, Lapsed P+ User, and Public Sector User, based on recent and historic condom brand use. Descriptive analyses on socio-demographic and brand pricing points were performed to assess overall brand dynamics. Additionally, opinions on statements related to brand equity, structured by commonly-used categories, were then dichotomized (agree/disagree). Outcomes of brand awareness, satisfaction/loyalty, perceived quality, leadership/popularity, perceived value, brand personality, and market barrier to acceptance were described and compared between the three groups.

Using confirmatory factor analysis procedures, factor loadings for variable in each brand equity construct were assessed, assuring adequate (>.5) individual factor loading and high overall (Chronbach's Alpha >0.6) inclusion between both questions asked concerning Protector Plus and public sector condoms. These thresholds for factor inclusion were chosen following the widely used Comrey and Lee (1992) criteria [16]. After assembling suitable factors, scale variables were created to represent each brand equity category. This data was used to create summary tables of brand equity across the three user groups, and also to conduct logistical regressions on different perceptions of brand equity, controlling for potential socio-demographic confounders.

Using brand pricing point data, additional logistical regression analyses were performed to assess brand equity perceptions among those willing to pay more (or less) for either Protector Plus or public sector condoms.

## Results

Table 1 provides a descriptive statistics for brand equity and market factors in the sample. Results have been summarized at the factor level for brand equity factors (representing the factor score for each factor). Respondents generally have higher brand equity in their own brand than in the other in-market brand (ie, P+ brand equity is higher for P+ users than for public sector users), as expected. Overall, P+ users' brand equity in P+ was somewhat higher than public sector users' brand equity in the public sector brand. Lapsed P+ users generally had somewhat lower brand equity in P+ than current P+ users, but much higher than their brand equity in the public sector brand.

**Table 1:** Protector Plus Users' Brand Equity Descriptive Statistics

What brand of condoms can you recall?	Current P+	95% CI - Low	95% CI - High	Lapsed P+	95% CI - Low	95% CI - High	Free/Gov't User	95% CI - Low	95% CI - High
**Protector Plus**	**98.8%**	97.9%	99.8%	**96.4%**	95.2%	97.5%	**60.2%**	58.7%	61.7%
**Free Men**	**49.1%**	48.4%	49.7%	**49.3%**	48.4%	50.1%	**98.1%**	96.2%	100.0%
**Care**	**19.2%**	18.8%	19.6%	**14.9%**	14.4%	15.3%	**10.7%**	10.0%	11.3%
**Free Women**	**10.0%**	9.7%	10.3%	**3.3%**	3.1%	3.5%	**4.9%**	4.4%	5.3%
**Durex**	**17.3%**	16.9%	17.7%	**12.0%**	11.5%	12.4%	**9.7%**	9.1%	10.3%
**Choice**	**3.1%**	2.9%	3.2%	**1.4%**	1.3%	1.6%	**2.9%**	2.6%	3.2%
**Brand Equity Factors (factor analysis)**									
**Satisfaction/loyalty**									
**Protector Plus**	**92.8%**	92.3%	93.3%	**82.3%**	81.7%	83.0%	**41.9%**	41.1%	42.7%
**Free/Government**	**36.0%**	35.6%	36.4%	**24.3%**	23.9%	24.6%	**88.3%**	87.2%	89.3%
**Perceived quality**									
**Protector Plus**	**87.0%**	86.7%	87.4%	**83.5%**	83.1%	83.9%	**61.3%**	60.7%	62.0%
**Free/Government**	**36.9%**	36.7%	37.2%	**30.1%**	29.8%	30.4%	**74.6%**	74.0%	75.3%
**Leadership/popularity**									
**Protector Plus**	**90.7%**	90.2%	91.3%	**78.5%**	77.9%	79.1%	**42.0%**	41.3%	42.8%
**Free/Government**	**89.5%**	89.1%	89.9%	**85.0%**	84.5%	85.5%	**45.3%**	44.7%	46.0%
**Perceived value**									
**Protector Plus**	**89.1%**	88.6%	89.6%	**84.5%**	83.9%	85.1%	**43.3%**	42.5%	44.1%
**Free/Government**	**40.9%**	40.5%	41.3%	**32.2%**	31.8%	32.6%	**84.9%**	83.9%	86.0%
**Brand personality**									
**Protector Plus**	**90.4%**	90.0%	90.8%	**82.3%**	81.9%	82.8%	**62.2%**	61.5%	62.9%
**Free/Government**	**53.4%**	53.1%	53.7%	**43.7%**	43.3%	44.0%	**83.8%**	83.1%	84.6%
**Market Factors**									
**Protector Plus**	**90.0%**	89.4%	90.7%	**74.9%**	74.2%	75.6%	**30.6%**	29.8%	31.3%
**Free/Government**	**18.3%**	17.8%	18.7%	**7.9%**	7.6%	8.3%	**31.4%**	30.3%	32.5%

We conducted confirmatory factor analysis (CFA) for the brand equity scales. As found in previous studies the 5 brand equity scales - satisfaction/loyalty, quality, leadership/popularity, value and personality - each represented a single factor. Because we expected different reactions to the P+ and public sector brands, we ran separate CFA for the 2 sample strata. In each analysis, all factor loadings and Chronbach alpha statistics were high and above threshold for acceptable scales [16]. The lowest alpha observed was .68 among P+ users for the brand loyalty scale and all others were .73 or higher.

Table 2 shows results of a multivariable logistic regression model in which brand equity is compared between P+ users and all other respondents for each of the brand equity factors. Odds ratios above 1 represent higher brand equity in the referent brand among P+ users compared to all others in the sample. Current P+ users have higher loyalty in P+ than others, and lower loyalty to the public sector brand than all others. Current P+ users have lower market barriers (price is self-reported as a lower barrier to purchasing condoms) than all others.

**Table 2:** Multivariable Logistic Regressions: Current P+ users compared to all others*

Brand equity factors	Current P+ vs. Others			
	P+ Brand Associations	p-value	Free/Government Brand Associations	p-value
**Satisfaction/loyalty**	**2.22**	**<0.001**	**0.53**	**0.015**
**Perceived quality**	0.73	0.236	1.44	0.275
**Leadership/popularity**	1.20	0.406	0.82	0.506
**Perceived value**	0.73	0.168	0.87	0.546
**Brand personality**	1.54	0.069	0.86	0.561
**Market Barriers**	**1.64**	**<0.001**		

*Co-variates include age, education, marriage status, and SES.

Table 3 shows results of a similar model in which brand equity is compared between lapsed P+ users (those who have used P+ in past year, but more than 3 months ago) and all other respondents for each of the brand equity factors. Lapsed users have lower loyalty to P+ than others, and higher market barriers (price is self-reported as a lower barrier to purchasing condoms) compared to all others in the sample. However, these associations are marginally significant (*p *< .10). These users have lower perceptions of popularity of public sector brand and higher associations with personality of the public sector. Thus they represent a group of individuals who might be convinced to switch back to using P+ given the right marketing and incentives.

**Table 3:** Multivariable Logistic Regressions: Lapsed P+ users compared to all others*

Brand equity factors	Lapsed P+ vs. Others			
	P+ Brand Associations	p-value	Free/Government Brand Associations	p-value
**Satisfaction/loyalty**	**0.66**	**0.053**	0.88	0.644
**Perceived quality**	1.01	0.970	0.74	0.395
**Leadership/popularity**	0.98	0.939	**0.43**	**0.007**
**Perceived value**	1.80	0.008	0.77	0.296
**Brand personality**	0.80	0.322	**1.79**	**0.037**
**Market Barriers**	**0.81**	**0.078**	n/a	

*Co-variates include age, education, marriage status, and SES.

Table 4 shows results of an ordered multivariable logistic regression model in which all members of the sample are included. Odds ratios above 1 indicate that respondents are willing to pay higher prices given higher brand equity in factor indicated in that table row. Respondents indicated they were generally willing to pay higher price for brand given higher brand equity. Current P+ users are willing to pay higher prices for condoms given higher brand loyalty, higher personality associations and lower market barriers. Public sector users are willing to pay more given higher loyalty, quality, and value.

**Table 4:** Willingness to Pay Higher Prices: Ordered Multivariable Logistic Regression (Sample Includes All Users)*

Brand equity factors	Protector Plus		Free/Government	
	Odds Ratio	p-value	Odds Ratio	p-value
Satisfaction/loyalty	**2.17**	**<0.001**	**1.68**	**0.038**
Perceived quality	0.84	0.434	**1.88**	**0.042**
Leadership/popularity	1.44	0.073	1.00	0.987
Perceived value	1.09	0.675	**3.08**	**<0.001**
Brand personality	**1.83**	**0.007**	1.20	0.480
Market Barriers	**1.70**	**<0.001**	n/a	

*Co-variates include age, education, marriage status, and SES

## Discussion

Overall, P+ currently has a strong brand equity and market position in the Zimbabwe condom market, with market share of 48% at the time of our study Declines in HIV rates reported in recent years may in part be due to changes in behavior including increased use of condoms [1,2]. However, economic and political uncertainty and the switch to a dollar-based economy make it imperative to continue efforts to effectively market P+ and grow the overall condom market by improving the positioning of socially marketed condom brands.

One important implication of this research is that there are opportunities to grow the total market by further building up equity in the P+ brand [6,12]. We observed only small and limited differences in brand equity associations between current P+ and lapsed P+ participants. Specifically, current P+ users have higher brand loyalty and lower perceived market barriers compared to all others and compared directly to lapsed P+ users. Lapsed P+ users have lower loyalty and report higher perceived market barriers than other respondents. Thus there may be an opportunity to increase total condom use in Zimbabwe by encouraging lapsed P+ users to 'switch' back to using P+ by influencing their perceptions of the P+ brand. There is potential to grow the total market by persuading lapsed P+ users who are currently not using condoms regularly to switch back to regular P+ use.

One long-term goal of the Total Market Approach is to create opportunities for private sector products to replace socially marketed and freely available condoms over time as economic conditions and consumer demand allow [17]. It may be that some public sector users could afford to use P+ or another brand. Their switching would help to grow the total market as they would then be paying for condoms and would be candidates to eventually adopt commercially marketed brands.

While brand equity in P+ was much lower among public sector users than among current P+ users and lapsed users, levels of brand equity in P+ were still substantial among this group. Over 60% of public sector users self-reported awareness of P+, and the brand leadership/popularity factor differed by only 3 percentage points (45.3% v. 42%) between P+ and the public sector brand among these users (see Table 1 for details). Other differences in brand equity between the 2 brands were much larger among this group, but brand equity in P+ was generally above 40% and in some cases much higher. Thus some of these users may also be willing to switch if their brand equity in P+ was raised through repositioning and effective P+ brand promotion and management over time.

While this research suggests that the total market for condoms in Zimbabwe could be increased through switching, the question remains whether users' income and economic conditions in the country would support an increased percentage of sold condoms. This study suggests that the answer is yes. Our analysis of self-reported willingness to pay for condoms suggests both 1) that some users who are currently not paying for condoms (lapsed P+ and public sector) are willing to pay, and 2) some current P+ users are willing to pay more for the brand than its current price of 10 cents for a pack of 3. Higher brand equity, especially the loyalty, quality, and value factors, is associated with higher willingness to pay for condoms among public sector and P+ users.

Thus price does not appear to be a major barrier to condom use in Zimbabwe. Rather it is perceptions of the condom product, as measured by brand equity, that appear to be a greater driver of preferences for condom use. As noted in Table 4, the market could be grown for some users, including those currently paying nothing, by charging at the current P+ price. Even at higher price point than current 10 cents, many users may be willing to pay for condoms given effective brand promotion and management and consequent higher levels of brand equity.

P+ as a brand, and condom use as a category of health behavior, appears positioned to grow in Zimbabwe. Clearly future economic conditions, resolution of outstanding political turmoil, and public perceptions of condoms as a protective health behavior and of the P+ brand of condoms will influence future growth in the market. Continued brand research is needed to evaluate changes in brand equity. The question remains how NGOs such as PSI-Zimbabwe should actively manage their condoms brands to grow the total market for condoms.

## Competing interests

The authors declare that they have no competing interests.

## Authors' contributions

WDE conceptualized the study, developed the analysis plan, supervised analysis, and wrote sections of the paper. NT contributed to the study design and analysis plan and edited the entire paper. KL edited the entire paper. JS conducted statistical analysis and wrote sections of the paper. All authors read and approved the final manuscript.
